# Specification of implementation interventions to address the cascade of HIV care and treatment in resource-limited settings: a systematic review

**DOI:** 10.1186/s13012-017-0630-8

**Published:** 2017-08-08

**Authors:** Matthew D. Hickey, Thomas A. Odeny, Maya Petersen, Torsten B. Neilands, Nancy Padian, Nathan Ford, Zachary Matthay, David Hoos, Meg Doherty, Chris Beryer, Stefan Baral, Elvin H. Geng

**Affiliations:** 1 Division of General Internal Medicine, San Francisco General Hospital, Department of Medicine, University of California, San Francisco (UCSF), San Francisco, CA USA; 2 Kenya Medical Research Institute, Nairobi, Kenya; 3 Department of Biostatistics and Epidemiology, School of Public Health, University of California, Berkeley, CA USA; 4 Center for AIDS Prevention Studies, Department of Medicine, UCSF, San Francisco, CA USA; 5 Department of HIV/AIDS, World Health Organization, Geneva, Switzerland; 6 School of Medicine, UCSF, San Francisco, CA USA; 7 Mailman School of Public Health, Columbia University, New York, NY USA; 8 Center for Public Health and Human Rights, Department of Epidemiology, Johns Hopkins Bloomberg School of Public Health, Baltimore, MD USA; 9 Division of ID HIV and Global Medicine, San Francisco General Hospital, Department of Medicine, UCSF, Building 80, 6th Floor, 1001 Potrero Avenue, San Francisco, CA 94110 USA

**Keywords:** HIV, Resource-limited settings, Cascade of care, Implementation science, Reporting

## Abstract

**Background:**

The global response to HIV has started over 18 million persons on life-saving antiretroviral therapy (ART)—the vast majority in low- and middle-income countries (LMIC)—yet substantial gaps remain: up to 40% of persons living with HIV (PLHIV) know their status, while another 30% of those who enter care are inadequately retained after starting treatment. Identifying strategies to enhance use of treatment is urgently needed, but the conceptualization and specification of implementation interventions is not always complete. We sought to assess the completeness of intervention reporting in research to advance uptake of treatment for HIV globally.

**Methods:**

We carried out a systematic review to identify interventions targeting the adult HIV care cascade in LMIC dating from 1990 to 2017. We identified components of each intervention as “intervention types” to decompose interventions into common components. We grouped “intervention types” into a smaller number of more general “implementation approaches” to aid summarization.

We assessed the reporting of six intervention characteristics adapted from the implementation science literature: the actor, action, action dose, action temporality, action target, and behavioral target in each study.

**Findings:**

In 157 unique studies, we identified 34 intervention “types,” which were empirically grouped into six generally understandable “approaches.” Overall, 42% of interventions defined the actor, 64% reported the action, 41% specified the intervention “dose,” 43% reported action temporality, 61% defined the action target, and 69% reported a target behavior. Average completeness of reporting varied across approaches from a low of 50% to a high of 72%. Dimensions that involved conceptualization of the practices themselves (e.g., actor, dose, temporality) were in general less well specified than consequences (e.g., action target and behavioral target).

**Implications:**

The conceptualization and Reporting of implementation interventions to advance treatment for HIV in LMIC is not always complete. Dissemination of standards for reporting intervention characteristics can potentially promote transparency, reproducibility, and scientific accumulation in the area of implementation science to address HIV in low- and middle-income countries.

**Electronic supplementary material:**

The online version of this article (doi:10.1186/s13012-017-0630-8) contains supplementary material, which is available to authorized users.

## Background

The global public health response to HIV has made highly efficacious antiretroviral therapy widely available in low- and middle-income countries (LMIC), but vulnerable health systems as well as social and structural barriers to patient engagement have limited full impact. Today, as many as 30% of HIV-infected persons in the more broadly generalized HIV epidemics across sub-Saharan Africa have not been tested: 10%-25%% of those found to be living with HIV have not enrolled in HIV care and an estimated 30% of those who have started treatment are not adequately retained in care [1, 2]. In order to meet the ambitious 90-90-90 targets set by Joint United Nations Programme on HIV and AIDS (UNAIDS) in 2014, there is an urgent need for research to identify implementation interventions to promote uptake and sustained use of antiretroviral treatment [3]. Diverse implementation strategies to enhance uptake of HIV treatment include use of peer and lay healthcare workers, community-based treatment strategies, integration of HIV with maternal health services, and mHealth approaches.

At present, however, consensus about conceptualization and reporting of implementation research does not yet fully exist, potentially undermining the reproducibility, transparency, and generalizability of research in this area. Guidance for how best to specify implementation interventions is emerging, but uptake of these practices among researchers addressing HIV in the global context is unknown [4]. In 2013, Proctor et al. suggested that all implementation interventions should state at a minimum: who carries out the intervention (i.e., the “actors”); the specific activities (i.e., “action”); the timing, frequency, and intensity of those activities (i.e., “dose,” “temporality”); the target of the described action (i.e., “action target”); and the targeted behavior on the pathway to a desired outcome (i.e., “implementation outcomes”). In 2014, Pinnock et al. suggested formal Standards for Reporting Implementation Studies of Complex Interventions (STaRI) [5]. Subsequently, a checklist to facilitate reporting titled Template for Intervention Description and Replication (TIDieR) has also been published [6]. Prior reviews of interventions for promoting uptake of HIV treatment have synthesized effects of broad categories of implementation interventions [7–12], but none explicitly address the completeness of intervention specification and reporting.

To appraise the level of reporting the implementation science literature seeking to enhance the use of antiretroviral therapy in LMIC, we carried out a systematic review. We define an implementation intervention as any intervention that seeks to improve uptake or sustained delivery of HIV care and treatment across any step of the HIV cascade of care. We sub-divide antiretroviral therapy implementation into the well-described set of discrete steps known as the HIV “Cascade of Care.” While Other reviews have summarized the effects of these interventions, we seek to describing the extent to which reports describe the nature of the intervention being implemented using an approach adapted from Proctor et al. We compared completeness of intervention reporting across different types of implementation interventions (e.g., mHealth, peer support) as well as by outcomes as defined by the particular step in the HIV care cascade (e.g., HIV testing, retention in care) that the intervention addressed.

## Methods

### Search strategy

We searched for studies of implementation interventions that were evaluated against a comparator and which targeted the adult HIV care cascade in low- and middle-income countries, as defined by the World Bank. The steps in the care and treatment cascade include HIV testing, linkage to care, staging for ART, retention in pre-ART care, initiation of ART, retention on ART, and ART medication adherence [13]. We considered HIV RNA suppression to be a surrogate outcome that combined information from multiple cascade steps, rather than a specific process itself and thus did not include studies that focused solely on HIV RNA suppression as the outcome. We excluded studies that merely evaluated the impact of predictors that are not directly modifiable on outcomes of interest (e.g., impact of gender or socioeconomic status on outcomes). We included prevention of mother to child transmission (PMTCT) studies that focused on maternal cascade outcomes, but to prevent excessive heterogeneity of patient populations, we excluded studies solely focused on infant diagnosis or prevention. Full search criteria can be found in Additional file 1.

We conducted our search within PubMed, Cochrane CENTRAL, WHO Global Health Library, SCOPUS, and Web of Science. Our original search was conducted on March 27, 2014, and included studies from 1996 through that date. We subsequently updated the searches on February 28, 2017, using only PubMed (PubMed yielded 94% of the total articles from the initial search). Our search strategy included four primary search terms linked by “AND”: (1) term indicating that study involved a comparator (e.g., randomized trial, cohort, prospective, relative risk); (2) term indicating LMIC; (3) term indicating that study involved HIV; and (4) term indicating that study involved implementation intervention OR cascade of care outcome. Additionally, we reviewed relevant systematic reviews and consulted experts in the field to identify additional articles that were not included (yield of 21 additional studies not identified in the search).

All studies underwent title review by one author (MDH); at which point, clearly irrelevant studies were screened out—generally because they were duplicate reports, represented basic science work, did not involve any comparison, did not involve a LMIC, or were not addressing an HIV cascade of care outcome. The remaining studies underwent title and abstract review, with full-text consultation when necessary, by two reviewers (MDH and TAO). Studies were included or excluded in the final review by consensus after independent review; discrepancies were resolved by discussion together with a third reviewer (EHG).

### Measurements

We captured both coded and unstructured data from each intervention using a Microsoft Access database including identifying information, study design, and level of intervention (e.g., system, organization, individual) (see Additional file 2 for data forms). For practical reasons, we considered each study report as the basic unit of analysis. We recorded the number of patients included in assessment of the cascade outcome of interest in each study. To approximate the frequency of reporting of negative studies, we recorded whether the study reported a positive effect for any of the cascade outcomes included, defined as a statistically significant improvement in any cascade outcome recorded.

We collected information on the “cascade of care” step addressed in each study and assessed for reporting on the behavioral target, action target, actor, action, dose, and temporality of each intervention using a pre-defined protocol (Additional file 3). These characteristics were adapted from the framework proposed by Proctor et al. [4] (Table 1). We perceived the “behavioral target” to be the link between the intervention action and the cascade step. Although the original Proctor framework used “implementation outcomes” in this step, we view many implementation outcomes as either behaviors or immediate antecedents of behavior (e.g., acceptability, adoption, sustainability) and therefore substitute the term “behavioral target” broadly to represent any behavior in health systems, organizations, providers, patients, and community members the intervention was meant to change. In turn, these behavioral targets are in general necessary but usually insufficient component causes of a cascade step.

**Table 1:** Intervention components

Intervention component	Description	Example
Actor	People or organization responsible for carrying out the designated intervention action	For example, in a peer support intervention, whether or not the peer is a person living with HIV him or herself is an important aspect of being a peer
Action	The specific set of steps required for carrying out the intervention	For example, a study quantifying the effect of a decentralized system vs a non-decentralized system may not specify how decentralization occurred.
Dose	The frequency with which intervention components are delivered to target population	For example, counseling interventions could vary by the duration of each session, the frequency that sessions are delivered, and the total number of sessions
Temporality	The timing of intervention action as related to other underlying processes	For an intervention﻿ to accelerate ART initiation: patients attending an HIV clinic undergo brief counseling and are offered to start ART on the date of the first clinic visit
Action target	The capability, motivation, or opportunity of an individual or organization which the action is intended to modify	HIV testing: First, the government launches a community-based HIV testing campaign. Second, an outreach team attached to the testing campaign offers community members transportation to the campaign on a free bus. Finally, a lottery is being held at the campaign and one person who receives HIV testing will win a bicycle at the health campaign. In this example, the action target of the campaign itself is that the intervention creates an *opportunity* for HIV testing. The action target of the free bus is the patient’s *capability* to attend the campaign. The action target of the lottery is the patient’s *motivation* to attend the campaign
Behavioral target	The particular behavior the intervention action is intended to elicit as a result of its action on the action target (i.e., modification of capability, motivation, or opportunity of the targeted individual or organization). This may be identical to the cascade outcome or may be an additional behavior proximal to the cascade outcome	ART initiation: an implementation intervention to address this cascade gap could act on a patient behavioral target to encourage them to make a *verbal request of ART* from providers once they know they are eligible. Another intervention could work on a behavioral target in the providers so that they *offer or prescribe* ART more readily

We next used Susan Michie’s Capability, Opportunity, Motivation, and Behavior (COM-B) framework to identify whether an action target was present and whether this action target was the capability, opportunity, or motivation of the agent for whom the intervention attempted to change behavior [14]. Though the original Proctor framework states that an action target should be specified according to “conceptual models of implementation,” we felt that the COM-B framework provided a sufficiently general definition of the determinants of behavior change that could be broadly applied. We considered an action target to be present if the study authors specified a hypothesized intervention impact on at least one of these domains for the person or entity for whom the intervention is designed to change behavior. We next determined whether the study provided details on the specific actions that were taken in the intervention to achieve the intended behavior change, as well as the frequency and intensity of this action (i.e., dose) and the relation of the timing of the action to underlying events (i.e., temporality). Finally, we assessed whether the study reported who the agent was that carried out the action. For all of the above components, we counted them as “present” if any aspect of the component was mentioned, regardless of the quality or level of detail in reporting of the component in question. For example, an intervention to enhance the cascade step of “ART initiation” might seek to increase health care provider prescription of ART (i.e., the behavioral target) by changing their motivation to do so (i.e., the action target) through opinion-leader (i.e., actor)-led training about the risks of delay (i.e., the action). It might be specified that teaching would be carried out for new providers at the time of hire (i.e., temporality) and reinforced at hour-long sessions quarterly (i.e., dose).

We did not attempt to evaluate the quality of reporting within each domain due to the inherent subjectivity of such an assessment and lack of clear framework for rating such quality. Full description of our measurement approach can be found in the study protocol in Additional file 2, and further examples can be seen in Table 2. It should be noted that though the initial Proctor framework also included “justification” of the approach chosen as a key factor that should be reported, we excluded this from our assessment due to the inherent subjectivity in qualitatively assessing whether or not appropriate justification was provided.

**Table 2:**
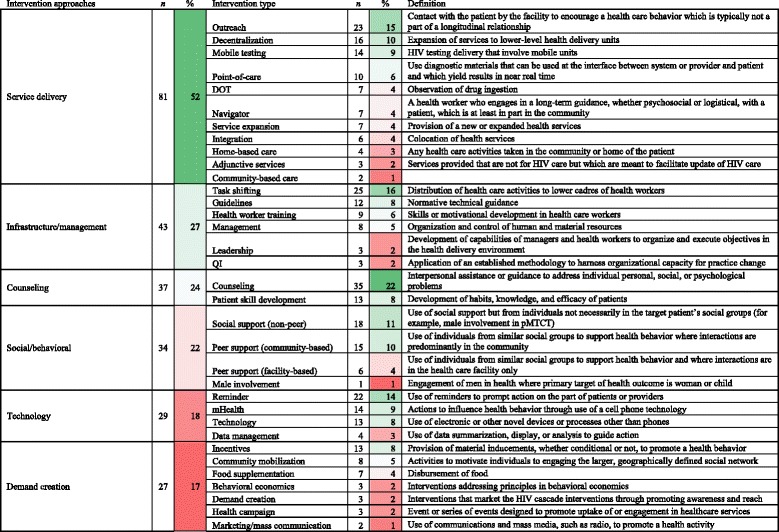
Frequency of intervention approaches and intervention types

### Analysis

#### Classification of interventions: intervention types and approaches

Implementation interventions reported in these studies were grouped in order to reduce the dimensionality of the data and facilitate summarization. The grouping was complicated by two facts: first, no two studies examined the exact same intervention and, second, many interventions were composed of multiple components, each with different actions. We attempted to summarize the types of interventions included in these studies using a two-step process. We first empirically classified each intervention using generally recognizable “prototypes” based largely on the action, agreed upon through iterative evaluation by three of the authors who work in the HIV field (MDH, TAO, EHG). We initially generated a list of all intervention types that these three authors had encountered in literature in the field. Subsequently, the above three authors independently extracted data from ten studies and, through discussion, developed consensus about classification of each study and intervention types included. Discussion of these articles led to additions to our list, after which we developed operational definitions for all intervention types included (Table 2). This list and set of definitions was used for full data extraction of all included articles. For example, we considered “counseling” to be an intervention type and defined this in accordance with common practice as “Interpersonal assistance or guidance to address individual personal, social or psychological problems.” Table 2 contains a full list intervention types utilized. Under this approach, a single intervention presented in a study report could be composed of multiple intervention types. For example, an intervention providing peer counseling with short message service (SMS) follow-up messages could be classified as “mHealth,” “counseling,” and “peer support.” Once we developed a full list of intervention types, the above authors empirically combined these interventions into six general groupings based on generally recognizable groupings of interventions. These more general groupings are referred to as “intervention approaches” (Table 2). The intervention types and approaches listed in Table 2 represent our best attempt at characterizing interventions, though it should be emphasized that misclassification is possible and reproducibility by others outside our group is not known.

#### Completeness of implementation intervention specification

We assessed the prevalence of reporting of the six characteristics adapted from Proctor overall, in each intervention approach, as well as for each step in the HIV cascade of care. We generated a suggested “score” from 0 to 6 for each study by summing the presence of reporting for each characteristic of interest. We then applied univariable linear regression to evaluate the association between study design, year of publication, intervention approach, and cascade step addressed on reporting completeness. We used robust standard errors to account for clustering within studies. To evaluate for nonlinear contributions of year of publication, we also fit restricted cubic splines and included them in an additional model.

## Results

### Search results and study characteristics

**Fig. 1:**
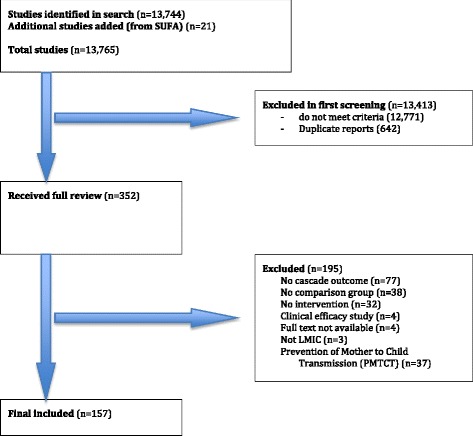
Results of systematic literature search

Our initial search yielded 13,744 articles (Fig. 1). Review of references from recent systematic reviews and consulting with experts in the field yielded an additional 21 articles that were included, resulting in 13,765 articles reviewed. Twelve thousand seven hundred seventy-one were excluded based on the titles (excluded if the title clearly suggested that the study was not LIMC, HIV-related, addressing a cascade of care outcome, or lacked a comparator group), and 642 were duplicate reports. Abstracts and, where necessary, full text of 352 articles was examined by both reviewers. Of these, 157 were included in the final analysis [15–171]. Eighty-eight percent of included interventions took place across sub-Saharan Africa (Table 3). The most common study designs were retrospective cohort studies (25%) and individual randomized controlled trials (25%), followed by before-and-after design (17%). Most interventions sought to influence individual patient behavior (63%, e.g., impact of peer-delivered directly observed therapy on individual patient adherence [133]) and non-patient community members (15%, e.g., encouraging community members to be tested for HIV [153]). The remaining studies addressed healthcare worker behavior (10%, e.g., task shifting intervention to allow nurses to initiate and refill ART [53]) and organizational behavior (13%, e.g., impact of guidelines on health center adherence to ART initiation within 8 weeks of TB treatment initiation [41]). The median sample size of included studies was 955 participants (IQR 400–4903).

**Table 3:** Study characteristics (*n* = 157)

	*n* (median where specified)	% (IQR where specified)
Study design		
Retrospective cohort	40	25
Individual RCT	40	25
Before-and-after	27	17
Cluster RCT	23	15
Prospective cohort	16	10
Quasi-experimental	7	4
Cross-sectional	2	1
Case-control	2	1
Region^a^		
Africa	138	87
Asia	13	8
Americas	7	4
Level of behavioral target^b^		
Individuals—patients	75	62
Individuals—community members	18	15
Organizations	15	12
Individuals—healthcare workers	12	10
Study reported a positive effect		
No	46	29
Yes	111	71
Year of publication		
2004	1	1
2005	0	0
2006	2	1
2007	4	3
2008	6	4
2009	7	4
2010	18	11
2011	17	11
2012	29	18
2013	18	11
2014	8	5
2015	15	10
2016	24	15
2017^c^	8	5
Sample size^d^ (median, IQR)	955	400 to 4903

^a^One study included sites in both Africa and Asia

^b^Limited to studies reporting a behavioral target (*n* = 120)

^c^Through 28 February 2017

^d^Sample size was determined by the number of individuals (patients or community members) included in the study, regardless of study design. Thus, sample size for cluster randomized controlled trials was recorded as the number of individuals, rather than the number of clusters

### Identification of intervention types and approaches

In the 157 studies, we identified a total of 34 intervention types (Table 2). The most common intervention type was counseling (22%), followed by task shifting (16%), outreach (15%), and reminders (14%). Most studies examined multi-faceted interventions. For example, Franke [60] reported on an “accompaniment” intervention in Rwanda where a community health worker made daily visits to the patients’ homes and provided social support, adverse event evaluation, directly observed therapy, and dispensed food supplementation. The intervention types therefore included actions of “counseling,” “social support (non-peer),” “directly observed therapy,” “food supplementation,” and “incentive.” As a point of contrast, Igumbor [77] studied a “patient advocate” intervention in South Africa in which facility-based staff made home visits, assessed barriers to adherence, counseled about adherence, and planned adherence support services. Therefore, this study included “counseling” and “peer support (community-based)” intervention types, but would not include “directly observed therapy” or “food supplementation.”

### Overall reporting of implementation interventions

**Fig. 2:**
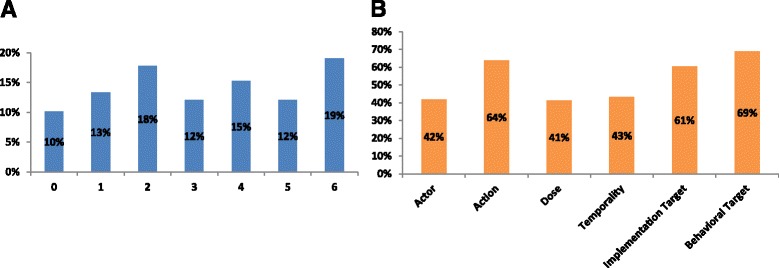
**a** Number of implementation parameters reported in each study (max 6, *N* = 157). **b** Prevalence of reporting of each implementation parameter in each study (*N* = 157)

Across all studies, the number of Proctor-based intervention dimensions reported was normally distributed with a median of three out of a maximum of six (Fig. 2). Ten percent (16/157) of studies reported zero of the six dimensions, and 19% (30/157) reported all six. In general, across all studies, the intervention dimensions that were closer to the cascade step of interest tended to be more completely reported than “upstream” components of the intervention (such as the actor, dose, and temporality): 69% of studies reported a “behavioral target” and 61% reported an “action target” of the intervention, whereas 64% reported the “action,” and 42% described “the actor” of the action (Additional file 4). Only 41% of studies reported the action “dose” and 43% reported “temporality.” In unadjusted linear regression, publication year was not associated with completeness of reporting (0.09 points per year, 95% CI −0.03 to 0.20, *p* = 0.14). Use of restricted cubic splines to model effect of publication year did not reveal any non-linear trend in reporting completeness either. Study design was associated with completeness of reporting. Compared to retrospective cohort studies (*n* = 40), most other study designs were associated with improved reporting completeness, with before-and-after design (*n* = 27, +1.7, 95% CI 0.82–2.7), individual randomized controlled trials (RCT) (*n* = 40, +2.0, 95% CI 1.1–2.8), and cluster RCTs (*n* = 23, +1.7, 95% CI 0.79–2.7) all reaching statistical significance, and quasi-experimental designs approaching significance (*n* = 7, +1.9, 95% CI −0.19 to 3.9).

### Intervention reporting by intervention approach

Overall, across six intervention approaches, the mean number of implementation dimensions reported ranged from 3.0 to 4.2 out of 6 (Table 4). Approaches that scored less well included service delivery (3.0), healthcare infrastructure/management (3.3), and social/behavioral (3.3). Technology interventions scored the highest (4.2). Examining the completeness of reporting of each Proctor dimension separately in each intervention approach provided additional resolution (Table 4). The actor was reported less than 50% of the time for demand creation, infrastructure/management, service delivery, and social/behavioral interventions. Dose and temporality were generally not well reported, with the exception of interventions using technology. Action target was also most frequently reported for technology interventions. The behavioral target was reported in more than 75% of the studies for four out of the six approaches. In a regression model, demand creation (+0.99, 95% CI 0.15–1.82) and technology interventions (1.42, 95% CI 0.69–2.15) were associated with more complete reporting (Table 5).

**Table 4:**
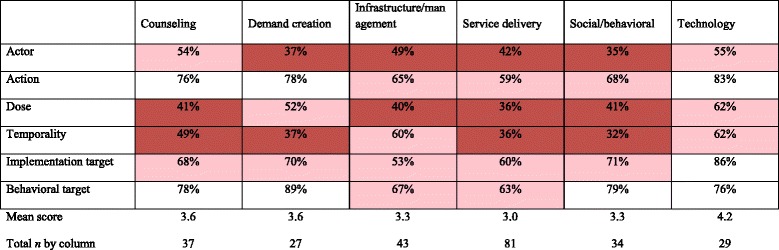
Completeness of reporting of Proctor-based intervention dimensions overall and by intervention approach

Cells in which reporting is less than 50% are dark red. Cells in which reporting is between 50 and 75% are pink. Cells in which reporting is above 75% are white. Totals are mean score out of total possible score of 6

**Table 5:** Association between implementation approach and interventions reporting

Implementation approach	Coefficient	95% CI		*p* value
Counseling	0.53	−0.14	−1.20	0.12
Demand creation	0.99	0.15	−1.82	0.02
Healthcare infrastructure/management	0.59	−0.21	−1.38	0.15
Service delivery	−0.17	−0.79	−0.45	0.58
Social/behavioral	−0.03	−0.74	−0.68	0.94
Technology	1.42	0.69	−2.15	<0.001

We used linear regression to assess change in a scale of one to six intervention dimensions reported on ten intervention approaches. Regression coefficients are interpreted as the change in score associated with the implementation approach

### Intervention reporting by cascade target

Reporting of intervention dimensions ranged from a low of 2.7 out of 6 (*n* = 56) for retention on ART to a high of 4.3 out of 6 for those targeting staging (*n* = 12) (Table 6). Actor and temporality was most infrequently reported in testing, retention on ART, and adherence interventions. Infrequent reporting of dose was also common for testing and retention on ART interventions, as well as initiation interventions. Interventions targeting post-ART retention showed the most incomplete reporting—less than 50% in four of the six dimensions. Adherence interventions reported the actor and temporality less than 50% but had similar reporting frequency to other cascade steps for the remaining categories. A behavioral target was reported in over 75% of studies for all cascade steps except for retention on ART. In linear regression of reporting completeness (on a scale of zero to six total Proctor-based dimensions) by cascade step as a categorical variable, interventions targeting adherence had a trend toward more complete reporting (+0.78, 95% CI −0.02 to 1.57, *p* = 0.06) (Table 7).

**Table 6:**
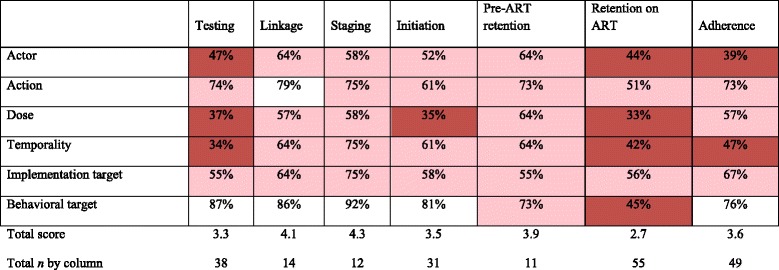
Completeness of reporting of Proctor-based intervention dimensions overall and by HIV care cascade step

Cells in which reporting is less than 50% are purple. Cells in which reporting is between 50 and 75% are pink. Cells in which reporting is above 75% are white

**Table 7:** Univariate regression of sum of six intervention reporting dimensions on each step in the cascade of care

Cascade step	Coefficient	95% CI		*p* value
Testing	0.32	−0.54	−1.18	0.47
Linkage to care	0.83	−0.37	−2.03	0.18
Staging	1.03	−0.23	−2.28	0.11
ART initiation	−0.02	−0.94	−0.90	0.96
Pre-ART retention in care	0.89	−0.63	−2.42	0.25
Retention in care on ART	−0.32	−1.09	−0.45	0.41
Adherence to ART	0.78	−0.02	−1.57	0.06

## Discussion

In this review, we found 157 studies that sought to improve uptake of HIV care and treatment in LMIC. We identified 34 intervention *types*, which we grouped into six general *approaches*. Overall, we found that implementation interventions addressing adult HIV care and treatment are often incompletely specified across dimensions that are important for fully characterizing a given intervention. A behavioral target for implementation interventions (the particular behavior that an intervention is intended to change) was specified with the greatest frequency, but even this dimension was reported in only two thirds of the studies, falling substantially short of universal coverage. Reporting of the “dose” and “temporality” of the particular intervention action were the least commonly reported.

We observed more complete reporting in certain types of interventions. Technological interventions, many of which were SMS-based interventions in this review, reported action, dose, temporality, and action target more consistently than other intervention approaches—a finding likely explained by the computerized, automated, and pre-programmed nature of systems to deliver SMS interventions. Despite increased frequency in reporting these domains, technology interventions reported a behavioral target with approximately the same frequency as other intervention approaches. The importance of identifying a behavioral target in an otherwise well-specified technology intervention is exemplified by a study in Kenya, which found that a weekly two-way SMS messaging system [102] improved virologic outcomes. However, the study did not report the intended behavioral or intervention targets, which could potentially help explain observed effects in both this and other subsequent studies of SMS, not all of which showed positive effects.  

Although multiple groups have issued reporting guidelines for implementation research [6], here, we extend the current scientific discourse on reporting through empiric quantification of the reporting gap in a specific topical area—care and treatment of HIV in LMIC. These findings suggest that implementation research targeting HIV treatment is an emerging area where standard reporting practices have not completely diffused into day-to-day scientific practice. Of concern, over the 13 years covered in this study, the average completeness of reporting has not changed.

Advancing reporting standards in research targeting implementation interventions is aligned with a broader movement in social and behavioral sciences to enhance transparency and bolster reproducibility [172]. One aspect of this movement is to ensure open access to materials that would be needed to reproduce the study; adequate specification of the details of the intervention is clearly critical to achieving such an objective. Specification also enables researchers seeking to evaluate the intervention in a new setting and implementers to scale up the intervention (perhaps with adaptations). For example, peer-based interventions where persons living with HIV and experienced with treatment offer knowledge, support, and care to those newly starting therapy are popular, but over 40% of such studies did not specify the selection, training, or remuneration of the peer educators under evaluation. Variation in the delivery of peer-based interventions along with the variable reporting are perhaps the two reasons that despite tremendous enthusiasm about their potential, some see peer-based interventions as nebulous and unconvincing.

Transportability—or the ability to use results from one setting to infer effects in another—takes a heightened importance for implementation interventions because contextual diversity in implementation environments is the rule. Work by Pearl et al. underscores the critical role of hypothesizing about and measuring the mechanisms of effect to make inferences about anticipated effects in another [173, 174]. For example, one included study of an opinion leader-led coaching intervention in Uganda sought to accelerate uptake of ART through influencing frontline healthcare workers (e.g., clinical officers, nurses) [20]. Qualitative work, however, revealed that healthcare workers (HCWs) in turn influenced peer health workers, who prepared patients for ART initiation in the community even before encountering formal HCW, thus catalyzing the ART initiation process through a mechanism outside of the original design. Understanding this mechanism suggests that this intervention might have diminished effects in settings without peer health workers and might be improved with formal incorporation of this cadre into the intervention design where peers do exist.

There are several limitations with the search protocol and analytic approaches reported here. First, there is no single search term that will consistently identify implementation interventions. In HIV care and treatment, however, there is a widely excepted heuristic (i.e., the “cascade of care”) for the macroscopic steps in public health activities (e.g., testing, linkage, retention, and adherence) that facilitated our search. Second, many interventions are composed of a “package” of different activities. We were unable to separate out intervention dimensions for each sub-component even though such an analysis might be revealing. Third, we grouped interventions into types and approaches that, by consensus of the authors, were understandable to public health practitioners; however, this grouping may be subject to debate and may not be comprehensive or reproducible. Fourth, deciding whether studies reported a particular characteristics of the implementation intervention is somewhat subjective, and therefore our assessments may be imperfectly reproducible. 

## Conclusions

Although intervention specification is critically important for pragmatic research, reporting of key intervention characteristics in studies targeting the HIV treatment cascade is not optimal. Poorly specified interventions present challenges to other researchers or to implementers who might seek to reproduce results or scale up the intervention, thus potentially undermining both scientific progress as well as real-world utility. Inadequately specified interventions also complicate informal as well as formal knowledge accumulation through meta-analyses, which hold great promise as a tool to extend comparative effectiveness research [175, 176]. There is a movement in the social sciences to promote transparency, yet incompletely specified interventions are, by definition, opaque. Improving specification of implementation interventions represents a core component of the process toward achieving sustained viral suppression for all those living with HIV to improve clinical outcomes and prevent onward HIV transmission.

## Additional files

Additional file 1: Search criteria. (DOCX 25 kb)

Additional file 2: Data entry form. (PDF 146 kb)

Additional file 3: Protocol. (DOCX 29 kb)

Additional file 4: Emblematic studies. (DOCX 15 kb)

## Abbreviations

ART: Antiretroviral therapy
COM-B: Capability, Opportunity, Motivation, and Behavior framework
HCW: Healthcare workers
HIV: Human immunodeficiency virus
LMIC: Low- and middle-income countries
PMTCT: Prevention of mother to child transmission
RNA: Ribonucleic acid
SMS: Short message service
STaRI: Standards for Reporting Implementation Studies of Complex Interventions
TIDieR: Template for Intervention Description and Replication
UNAIDS: Joint United Nations Programme on HIV and AIDS
WHO: World Health Organization
