# Supplementary material for: Specification of implementation interventions to address the cascade of HIV care and treatment in resource-limited settings: a systematic review
Source: Implement Sci. 2017 Aug 8;12:102. doi: 10.1186/s13012-017-0630-8 (PMC5547499; doi:10.1186/s13012-017-0630-8)
Supplement: Supplementary file 1 — Search criteria. (DOCX 25 kb) [file 13012_2017_630_MOESM1_ESM.docx]

**Search Criteria**

*Supplemental Material*

We conducted our search within Pubmed, Cochrane CENTRAL, WHO Global Health Library, SCOPUS, and Web of Science. Our search strategy included four primary search terms linked by ‘AND’: 1) term indicating study involved a comparator (e.g. randomized trial, cohort, prospective, relative risk, etc); 2) term indicating low or middle income country (LMIC); 3) term indicating the study involved HIV; and 4) term indicating that study involved implementation intervention OR cascade of care outcome. We restricted studies to 1996 through 2014. Additionally, we reviewed relevant systematic reviews and consulted experts in the field to identify additional articles that were not included (yield of 21 additional studies not identified in search). Overall, we yielded 9955 studies, including 8805 studies from Pubmed, 995 from Cochrane CENTRAL, 127 from WHO Global Health Library, 367 from SCOPUS, 878 from Web of Science, and 21 from systematic review and expert consultation.

All studies underwent title review by one author (MDH), at which point 9109 clearly irrelevant studies were screened out – generally because they represented duplicate reports, basic science work, did not involve any comparison, or did not involve a LMIC. The remaining 235 studies underwent title and abstract review, with full text consultation when necessary, by two reviewers (MDH and TAO). Studies were included or excluded in the final review by consensus after independent review; discrepancies were resolved by discussion together with a third reviewer (EHG). One hundred and sixty three (163) studies proceeded to full data abstraction following this review, though during this process an additional 57 studies were excluded because they did not meet inclusion criteria. One hundred and six studies were included in the final analysis.

See below for full description of search terms.

**Review of primary studies**

**Total references n=9934 (original search 3/2014)**

**Revised search 2/28/2017: 3810 additional articles using Pubmed search below**

**Core PubMed strategy**

| Search | Query | Items found |
| --- | --- | --- |
| **#6** | **Publication date from 1996/01/01 to 2014/12/31** | **8805** |
| #5 | #1 AND #2 AND #3 AND #4 | 9443 |
| #4 | Search randomized controlled trial[pt] OR controlled clinical trial[pt] OR randomized controlled trials[mh] OR random allocation[mh] OR double-blind method[mh] OR single-blind method[mh] OR clinical trial[pt] OR clinical trials[mh] OR (clinical trial[tw]) OR ((singl*[tw] OR doubl*[tw] OR trebl*[tw] OR tripl*[tw]) AND (mask*[tw] OR blind*[tw])) OR placebos[mh] OR placebo*[tw] OR random*[tw] OR non-random*[tw] OR before after study[tw] OR time series[tw] OR case control[tw] OR prospective*[tw] OR retrospective*[tw] OR cohort[tw] OR cross-section*[tw] OR prospective[tw] OR retrospective[tw] OR research design [mh:noexp] OR comparative study[mh] OR evaluation studies[mh] OR follow-up studies[mh] OR prospective studies[mh] OR control*[tw] OR prospectiv*[tw] OR volunteer*[tw] OR longitud*[tw] OR descripti*[tiab] OR study[tiab] OR evaluat*[tiab] OR odds ratio[tw] OR hazard ratio[tw] OR relative risk[tw] OR risk ratio[tw] OR rate ratio[tw] OR AOR[tw] OR RRR[tw] OR NNT[tw]) | 9018202 |
| #3 | Search Afghanistan[tiab] OR Albania[tiab] OR Algeria[tiab] OR American Samoa[tiab] OR Angola[tiab] OR Antigua and Barbuda[tiab] OR Argentina[tiab] OR Armenia[tiab] OR Azerbaijan[tiab] OR Bangladesh[tiab] OR Belarus[tiab] OR Belize[tiab] OR Benin[tiab] OR Bhutan[tiab] OR Bolivia[tiab] OR Bosnia and Herzegovina[tiab] OR Botswana[tiab] OR Brazil[tiab] OR Bulgaria[tiab] OR Burkina Faso[tiab] OR Burundi[tiab] OR Cambodia[tiab] OR Cameroon[tiab] OR Cabo Verde[tiab] OR Central African Republic[tiab] OR Chad[tiab] OR Chile[tiab] OR China[tiab] OR Colombia[tiab] OR Comoros[tiab] OR Congo[tiab] OR Costa Rica[tiab] OR Côte d'Ivoire[tiab] OR Cuba[tiab] OR Djibouti[tiab] OR Dominica[tiab] OR Dominican Republic[tiab] OR Ecuador[tiab] OR Egypt[tiab] OR El Salvador[tiab] OR Eritrea[tiab] OR Ethiopia[tiab] OR Fiji[tiab] OR Gabon[tiab] OR Gambia[tiab] OR Georgia[tiab] OR Ghana[tiab] OR Grenada[tiab] OR Guatemala[tiab] OR Guinea[tiab] OR Guinea-Bissau[tiab] OR Guyana[tiab] OR Haiti[tiab] OR Honduras[tiab] OR India[tiab] OR Indonesia[tiab] OR Iran[tiab] OR Iraq[tiab] OR Jamaica[tiab] OR Jordan[tiab] OR Kazakhstan[tiab] OR Kenya[tiab] OR Kiribati[tiab] OR North Korea [tiab] OR Kosovo[tiab] OR Kyrgyz [tiab] OR Lao[tiab] Laos[tiab] OR Latvia[tiab] OR Lebanon[tiab] OR Lesotho[tiab] OR Liberia[tiab] OR Libya[tiab] OR Lithuania[tiab] OR Macedonia[tiab] OR Madagascar[tiab] OR Malawi[tiab] OR Malaysia[tiab] OR Maldives[tiab] OR Mali[tiab] OR Marshall Islands[tiab] OR Mauritania[tiab] OR Mauritius[tiab] OR Mexico[tiab] OR Micronesia[tiab] OR Moldova[tiab] OR Mongolia[tiab] OR Montenegro[tiab] OR Morocco[tiab] OR Mozambique[tiab] OR Myanmar[tiab] OR Namibia[tiab] OR Nepal[tiab] OR Nicaragua[tiab] OR Niger[tiab] OR Nigeria[tiab] OR Pakistan[tiab] OR Palau[tiab] OR Panama[tiab] OR Papua New Guinea[tiab] OR Paraguay[tiab] OR Peru[tiab] OR Philippines[tiab] OR Romania[tiab] OR Russia[tiab] OR Russian Federation[tiab] OR Rwanda[tiab] OR Samoa[tiab] OR Sao Tome[tiab] OR Senegal[tiab] OR Serbia[tiab] OR Seychelles[tiab] OR Sierra Leone[tiab] OR Solomon Islands[tiab] OR Somalia[tiab] OR South Africa[tiab] OR South Sudan[tiab] OR Sri Lanka[tiab] OR St. Lucia[tiab] OR St. Vincent[tiab] OR Grenadines[tiab] OR Sudan[tiab] OR Suriname[tiab] OR Swaziland[tiab] OR Syrian Arab Republic[tiab] OR Tajikistan[tiab] OR Tanzania[tiab] OR Thailand[tiab] OR Timor-Leste[tiab] OR Togo[tiab] OR Tonga[tiab] OR Tunisia[tiab] OR Turkey[tiab] OR Turkmenistan[tiab] OR Tuvalu[tiab] OR Uganda[tiab] OR Ukraine[tiab] OR Uruguay[tiab] OR Uzbekistan[tiab] OR Vanuatu[tiab] OR Venezuela[tiab] OR Vietnam[tiab] OR West Bank[tiab] OR Gaza[tiab] OR Yemen[tiab] OR Zambia[tiab] OR Zimbabwe [tiab] OR “developing countries”[tiab] OR “resource-limited”[tiab] OR “resource-constrained”[tiab] OR “low- and middle-income”[tiab] OR LMIC[tiab] OR “third world”[tiab] OR “low income countries”[tiab] | 277217 |
| #2 | Search HIV Infections[MeSH] OR HIV[MeSH] OR hiv[tw] OR hiv-1*[tw] OR hiv-2*[tw] OR hiv1[tw] OR hiv2[tw] OR hiv infect*[tw] OR human immunodeficiency virus[tw] OR human immune deficiency virus[tw] OR human immuno-deficiency virus[tw] OR human immune-deficiency virus[tw] OR ((human immun*) AND (deficiency virus[tw])) OR acquired immunodeficiency syndrome[tw] OR acquired immunodeficency syndrome[tw] OR acquired immuno-deficiency syndrome[tw] OR acquired immune-deficiency syndrome[tw] OR ((acquired immun*) AND (deficiency syndrome[tw])) OR "sexually transmitted diseases, viral"[MESH:NoExp] | 315090 |
| #1 | Search AIDS Serodiagnosis/organization and administration[Mesh] OR (Test*[tiab] AND (volunt*[tiab] OR counsel*[tiab] OR VCT[tiab] OR HCT[tiab] OR HTC[tiab] OR PITC[tiab] OR provider-initiated[tiab] OR home[tiab] OR homes[tiab] OR homebased[tiab] OR home-based[tiab] OR homecare[tiab] OR home-care[tiab] OR home access[tiab] OR in-home[tiab] OR domicile*[tiab] OR facility-based[tiab] OR facilitybased[tiab] OR door to door[tiab] OR door-to-door[tiab])) **OR** (HAART[tiab] OR ART[tiab] OR ARV[tiab] OR ARVs[tiab] OR cART[tiab] antiretroviral[tiab] OR anti-retroviral[tiab] OR anti-viral[tiab] OR antiviral[tiab] OR Antiretroviral Therapy, Highly Active[Mesh]) **OR** (link*[tiab] OR enrol* OR initiat* OR eligib*[tiab] OR begin*[tiab] OR enter*[tiab] OR start*[tiab]) **OR** (retention[tiab] OR retain*[tiab] OR lost to follow-up [tiab] OR loss to follow-up [tiab] OR (loss*[tiab] AND follow up [tiab]) OR LTFU[tiab] OR attrition[tiab] OR loss to care [tiab] OR lost to care [tiab] OR loss to program [tiab] OR lost to program [tiab] OR default*[tiab] OR engage*[tiab] OR disengage*[tiab])) **OR** (Medication Adherence[Mesh] OR adherence[ti] OR compliance[ti]) OR (viral load[tiab] OR virologic*[tiab] OR suppress*[tiab] OR polymerase[tiab] OR PCR[tiab] OR RNA[tiab]) | 3267771 |

**Cochrane CENTRAL (n=995 refs)**

'HIV* OR AIDS OR human immunodeficiency OR acquired immunodeficiency OR acquired immune deficiency OR human immune deficiency **in Record Title and**

Afghanistan OR Albania OR Algeria OR American Samoa OR Angola OR Antigua and Barbuda OR Argentina OR Armenia OR Azerbaijan OR Bangladesh OR Belarus OR Belize OR Benin OR Bhutan OR Bolivia OR Bosnia and Herzegovina OR Botswana OR Brazil OR Bulgaria OR Burkina Faso OR Burundi OR Cambodia OR Cameroon OR Cabo Verde OR Central African Republic OR Chad OR Chile OR China OR Colombia OR Comoros OR Congo OR Costa Rica OR Côte d'Ivoire OR Cuba OR Djibouti OR Dominica OR Dominican Republic OR Ecuador OR Egypt OR El Salvador OR Eritrea OR Ethiopia OR Fiji OR Gabon OR Gambia OR Georgia OR Ghana OR Grenada OR Guatemala OR Guinea OR Guinea-Bissau OR Guyana OR Haiti OR Honduras OR India OR Indonesia OR Iran OR Iraq OR Jamaica OR Jordan OR Kazakhstan OR Kenya OR Kiribati OR North Korea OR Kosovo OR Kyrgyz OR Lao Laos OR Latvia OR Lebanon OR Lesotho OR Liberia OR Libya OR Lithuania OR Macedonia OR Madagascar OR Malawi OR Malaysia OR Maldives OR Mali OR Marshall Islands OR Mauritania OR Mauritius OR Mexico OR Micronesia OR Moldova OR Mongolia OR Montenegro OR Morocco OR Mozambique OR Myanmar OR Namibia OR Nepal OR Nicaragua OR Niger OR Nigeria OR Pakistan OR Palau OR Panama OR Papua New Guinea OR Paraguay OR Peru OR Philippines OR Romania OR Russia OR Russian Federation OR Rwanda OR Samoa OR Sao Tome OR Senegal OR Serbia OR Seychelles OR Sierra Leone OR Solomon Islands OR Somalia OR South Africa OR South Sudan OR Sri Lanka OR St. Lucia OR St. Vincent OR Grenadines OR Sudan OR Suriname OR Swaziland OR Syrian Arab Republic OR Tajikistan OR Tanzania OR Thailand OR Timor-Leste OR Togo OR Tonga OR Tunisia OR Turkey OR Turkmenistan OR Tuvalu OR Uganda OR Ukraine OR Uruguay OR Uzbekistan OR Vanuatu OR Venezuela OR Vietnam OR West Bank OR Gaza OR Yemen OR Zambia OR Zimbabwe OR "developing countries" OR "resource-limited" OR "resource-constrained" OR "low- and middle-income" OR LMIC OR "third world" OR "low income countries" **in Title, Abstract, Keywords and**

Test* AND volunt* OR counsel* OR VCT OR HCT OR HTC OR PITC OR provider-initiated OR home OR homes OR homebased OR home-based OR homecare OR home-care OR home access OR in-home OR domicile* OR facility-based OR facilitybased OR door to door OR door-to-door OR HAART OR ART OR ARV OR ARVs OR cART antiretroviral OR anti-retroviral OR anti-viral OR antiviral OR link* OR enrol* OR initiat* OR eligib* OR begin* OR enter* OR start* OR retention OR retain* OR lost to follow-up OR loss to follow-up OR loss* AND follow up OR LTFU OR attrition OR loss to care OR lost to care OR loss to program OR lost to program OR default* OR engage* OR disengage* OR adherence OR compliance OR viral load OR virologic* OR suppress* OR polymerase OR PCR OR RNA **in Title, Abstract, Keywords**

**WHO Global Health Library (n=127)**

(all fields)

HIV* AND (Test* AND volunt* OR counsel* OR VCT OR HCT OR HTC OR PITC OR provider-initiated OR home OR homes OR homebased OR home-based OR homecare OR home-care OR home access OR in-home OR domicile* OR facility-based OR facilitybased OR door to door OR door-to-door OR HAART OR ART OR ARV OR ARVs OR cART antiretroviral OR anti-retroviral OR anti-viral OR antiviral OR link* OR enrol* OR initiat* OR eligib* OR begin* OR enter* OR start* OR retention OR retain* OR lost to follow-up OR loss to follow-up OR loss* AND follow up OR LTFU OR attrition OR loss to care OR lost to care OR loss to program OR lost to program OR default* OR engage* OR disengage* OR adherence OR compliance OR viral load OR virologic* OR suppress* OR polymerase OR PCR OR RNA)

**SCOPUS including Embase (n=367)**

(ALL(hiv* OR aids OR human immunodeficiency OR acquired immunodeficiency OR acquired immune deficiency OR human immune deficiency)) AND (ALL(test* AND volunt* OR counsel* OR vct OR hct OR htc OR pitc OR provider-initiated OR home OR homes OR homebased OR home-based OR homecare OR home-care OR home access OR in-home OR domicile* OR facility-based OR facilitybased OR door to door OR door-to-door OR haart OR art OR arv OR arvs OR cart antiretroviral OR anti-retroviral OR anti-viral OR antiviral OR link* OR enrol* OR initiat* OR eligib* OR begin* OR enter* OR start* OR retention OR retain* OR lost to follow-up OR loss to follow-up OR loss* AND follow up OR ltfu OR attrition OR loss to care OR lost to care OR loss to program OR lost to program OR default* OR engage* OR disengage* OR adherence OR compliance OR viral load OR virologic* OR suppress* OR polymerase OR pcr OR rna)) AND (LIMIT-TO(SUBJAREA, "MEDI") OR LIMIT-TO(SUBJAREA, "SOCI"))

**Web of Science (n=878)**

**TITLE:** (HIV* OR AIDS OR human immunodeficiency OR acquired immunodeficiency OR acquired immune deficiency OR human immune deficiency) AND **TITLE:** (Afghanistan OR Albania OR Algeria OR American Samoa OR Angola OR Antigua and Barbuda OR Argentina OR Armenia OR Azerbaijan OR Bangladesh OR Belarus OR Belize OR Benin OR Bhutan OR Bolivia OR Bosnia and Herzegovina OR Botswana OR Brazil OR Bulgaria OR Burkina Faso OR Burundi OR Cambodia OR Cameroon OR Cabo Verde OR Central African Republic OR Chad OR Chile OR China OR Colombia OR Comoros OR Congo OR Costa Rica OR Côte d'Ivoire OR Cuba OR Djibouti OR Dominica OR Dominican Republic OR Ecuador OR Egypt OR El Salvador OR Eritrea OR Ethiopia OR Fiji OR Gabon OR Gambia OR Georgia OR Ghana OR Grenada OR Guatemala OR Guinea OR Guinea-Bissau OR Guyana OR Haiti OR Honduras OR India OR Indonesia OR Iran OR Iraq OR Jamaica OR Jordan OR Kazakhstan OR Kenya OR Kiribati OR North Korea OR Kosovo OR Kyrgyz OR Lao Laos OR Latvia OR Lebanon OR Lesotho OR Liberia OR Libya OR Lithuania OR Macedonia OR Madagascar OR Malawi OR Malaysia OR Maldives OR Mali OR Marshall Islands OR Mauritania OR Mauritius OR Mexico OR Micronesia OR Moldova OR Mongolia OR Montenegro OR Morocco OR Mozambique OR Myanmar OR Namibia OR Nepal OR Nicaragua OR Niger OR Nigeria OR Pakistan OR Palau OR Panama OR Papua New Guinea OR Paraguay OR Peru OR Philippines OR Romania OR Russia OR Russian Federation OR Rwanda OR Samoa OR Sao Tome OR Senegal OR Serbia OR Seychelles OR Sierra Leone OR Solomon Islands OR Somalia OR South Africa OR South Sudan OR Sri Lanka OR St. Lucia OR St. Vincent OR Grenadines OR Sudan OR Suriname OR Swaziland OR Syrian Arab Republic OR Tajikistan OR Tanzania OR Thailand OR Timor-Leste OR Togo OR Tonga OR Tunisia OR Turkey OR Turkmenistan OR Tuvalu OR Uganda OR Ukraine OR Uruguay OR Uzbekistan OR Vanuatu OR Venezuela OR Vietnam OR West Bank OR Gaza OR Yemen OR Zambia OR Zimbabwe OR "developing countries" OR "resource-limited" OR "resource-constrained" OR "low- and middle-income" OR LMIC OR "third world" OR "low income countries") AND **TOPIC:** (random* OR cohort*) AND **TOPIC:** (eligib* OR link* OR referr* OR lost OR loss OR initia* OR retention OR LTFU) NOT **TITLE:** (prevent*)

Timespan=All years. Indexes=SCI-EXPANDED, SSCI, A&HCI, CPCI-S, CPCI-SSH, BKCI-S, BKCI-SSH, CCR-EXPANDED, IC.
