# Supplementary material for: Specification of implementation interventions to address the cascade of HIV care and treatment in resource-limited settings: a systematic review
Source: Implement Sci. 2017 Aug 8;12:102. doi: 10.1186/s13012-017-0630-8 (PMC5547499; doi:10.1186/s13012-017-0630-8)
Supplement: Supplementary file 2 — Data entry form. (PDF 146 kb) [file 13012_2017_630_MOESM2_ESM.pdf]

First author | Year | Journal
Title
Citation

NEW STUDY | **Study excluded** PMTCT ☐ Does not meet criteria ☐ Reason | **Delete Study**

Characteristics | Proctor

Study data abstraction complete? ☐

Publication type
Study design
Design other
Country
Region

Setting
The context where the action is carried out.

Clinic or Community?
Urban or Rural?

Inclusion/exclusion criteria

Intervention

Comparator

Comments

Sample size

Did the study have a positive effect? ○ Yes ○ No

Key Populations Addressed

	Yes	No
MSM	○	○
PWID	○	○
Pregnant women	○	○
TB	○	○
Infants	○	○
Children	○	○
Orphans	○	○
Street children	○	○
Adolescents	○	○
CSW	○	○
Disabled	○	○
Prisoners	○	○
Migrants	○	○

Key pop comments

First author | Year | Journal

Title

Citation

NEW STUDY

Study excluded PMTCT ☐ Does not meet criteria ☐ Reason

Delete Study

Characteristics | Proctor

Reviewer | Proctor review complete? ☐

Delete Proctor entry

Cascade step(s) targeted

	Yes	No
Testing	○	○
Linkage	○	○
Staging	○	○
Pre-ART retention	○	○
Initiation	○	○
ART retention	○	○
Adherence	○	○

Comments:

Behavioral target

Note: behavioral target may or may not be the same as the cascade step. Mediator is any behavioral target proported to mediate intervention effect on cascade outcome.

Behavioral target specified ○ Yes ○ No

Target is separate from cascade ○ Yes ○ No

Level of behavioral target

Describe behavioral target:

Implementation intervention target

Implementation target specified ○ Yes ○ No

COM components present:

Capability ○ Yes ○ No

Opportunity ○ Yes ○ No

Motivation ○ Yes ○ No

Comment

Actor-Action

Action specified ◯ Yes ◯ No

Action description:

Actor specified ◯ Yes ◯ No

Dose specified ◯ Yes ◯ No

Temporality specified ◯ Yes ◯ No

Intervention classification

Intervention 'named' ◯ Yes ◯ No

Intervention Type

Implementation measures

Any implementation measure ◯ Yes ◯ No

- Feasibility ◯ Yes ◯ No
- Acceptability ◯ Yes ◯ No
- Adoption ◯ Yes ◯ No
- Fidelity ◯ Yes ◯ No
- Cost ◯ Yes ◯ No
- Sustainability ◯ Yes ◯ No

Comment

Implementation outcomes

Any COM implementation outcome ◯ Yes ◯ No

- Capability ◯ Yes ◯ No
- Opportunity ◯ Yes ◯ No
- Motivation ◯ Yes ◯ No

Behavioral outcome ◯ Yes ◯ No

Relevant only if behavioral target is separate from cascade

Comment

Record: 1 of 1 No Filter Search
