# Supplementary material for: Specification of implementation interventions to address the cascade of HIV care and treatment in resource-limited settings: a systematic review
Source: Implement Sci. 2017 Aug 8;12:102. doi: 10.1186/s13012-017-0630-8 (PMC5547499; doi:10.1186/s13012-017-0630-8)
Supplement: Supplementary file 3 — Protocol. (DOCX 29 kb) [file 13012_2017_630_MOESM3_ESM.docx]

**Evaluating Implementation Interventions using a Framework Derived from “Implementation strategies: recommendations for specifying and reporting” (Proctor et al. Implementation Science 2013, 8:139)**

**May 20, 2015**

**Background**

Global and national investments have made antiretroviral treatment for HIV/AIDS – which is highly efficacious – widely available in low and middle income countries, particularly in Africa, where the disease burden is the highest. Achievement, however, of the macroscopic steps in a public health response to make use of antiretroviral therapy, including HIV testing, linkage to care for those testing positive, treatment initiation for eligible patients and retention are far from optimal – some estimates suggest that only 25% of HIV infected persons have received and sustained treatment in low and middle income countries.

Identifying generalizable implementation interventions to enhance uptake of the cascade of care is critical for progress against HIV. Because implementation strategies seek to change complex behaviors – of systems, organizations, communities, patients, or providers – the interventions themselves can be complex, adaptive, composed of multiple components – and therefore challenging to fully specify in a standardized terms. Inadequate specification, however, undermines transparency, reproducibility and summarization in systematic reviews and meta-analysis and therefore the overall process of scientific accumulation.

To quantify the “reporting gap,” we conducted a systematic review to identify papers reporting on implementation interventions targeting the uptake of HIV care and treatment services in resource limited settings. We apply a modification of Proctor’s proposed dimensions. Proctor et al., as well as other groups such Standard for reporting Implementation Studies (StaRI) to quantify shortcomings in reporting and explore associations between intervention types or cascade targets and completeness of reporting. We examine published manuscripts that compare an intervention against a comparator group and describe a “backwards” process where we start by identify the cascade steps targeted by the intervention. The cascade steps are (1) HIV testing, (2) linkage to care among those testing positive; (3) clinical staging to determine eligibility for treatment among those who link to care; (4) ART initiation among patients found eligible; (5) retention among those on ART as well as those found not eligible initially. We use definitions of each of these steps as presented by authors – recognizing there is heterogeneity in definitions especially in those steps that occur over time such as retention.

The implementation characteristics we seek to document in each study are taken largely from Proctor et al. We seek to specify whether paper’s report an (1) actor, (2) action, (3) action dose, (4) action temporality, (5) action target, (6) implementation outcome and (7) the cascade step. We modify Proctor’s approach slightly by summarizing across all implementation outcomes through an generic category of a “behavioral target” which captures diverse behavioral consequences of actions that mediate the effects of actions on occurrence of a particular cascade step (e.g., HIV testing). We note the presence or absence of each characteristic without further quantification of the “quality” of specification. Many studies present interventions with multiple components – each in theory could have separate actors, actions, etc. We do not attempt to dissect multi-component interventions into constituent parts and assess Proctor dimensions in each part (which in theory could be done). Rather we accept the intervention package as presented by authors as a single intervention – in which we “give credit” for the specification of any one actor or action even if there could be several.

**Identification of Proctor Dimensions**

1. **Step 1: Identify the HIV care cascade step(s) targeted by the implementation intervention**
   1. **Completing steps in the HIV cascade of care is the goal of implementation interventions to advance the public health response to HIV.**

The HIV field has come to use the “cascade of care” to understand the major health care steps needed for individuals to benefit from clinical interventions to treat HIV. These steps are HIV testing, linkage of HIV infected persons to clinical care, staging and evaluation for ART eligibility (in areas where treatment depends on CD4 levels or clinical diagnoses), retention in care between linkage and ART initiation, ART initiation for eligible patients, retention on HIV treatment after treatment initiation, and medication adherence. Although popular representations of the cascade typically include viral suppression as the last step, we feel that viral suppression is a “health status outcome,” whereas these preceding steps are “benchmark” processes in the delivery of HIV care and therefore the immediate targets of implementation interventions. This heuristic is so penetrant in the HIV literature that we anticipate virtually all implementation interventions to target one of these steps.

1. **Step 2: Identify whether the behavioral target** **of the implementation intervention that influences completion of each of the cascade steps was specified in the manuscript.**
   1. **Implementation interventions drive the occurrence of an HIV care cascade step through changing a particular target behavior.**

We perceived this step to be the link between the action and the cascade step. Although the original Proctor framework used “implementation outcomes” in this step, we view many of the implementation outcomes as either behaviors or immediate antecedents of behavior (e.g., acceptability, adoption, sustainability) and therefore use behavioral target broadly to represent any behavior in health systems, organizations, provider, patients and community members the intervention was meant to change. In turn, these behavioral targets are in general necessary but usually insufficient component causes of a cascade step.

- - 1. **Example: behavioral target for HIV testing**

An implementation intervention of perhaps a petition could for example target the behavior of legislators and bureaucrats in the Ministry of Health in Uganda to *vote* in favor of a proposal to allocate funds for “mobile treatment delivery” units – which could increase uptake of HIV treatment by reaching patients who reside in geographically remote areas. Alternatively if these mobile units already exist, another implementation intervention could be a lottery for enrollment of unregistered patients into mobile clinics. In this intervention, *attending* the mobile service by the patient is the behavioral target of the lottery.

- - 1. **Example: behavioral target for ART adherence**

Intervention such as an alarm or a pill boxes have been used to enhance patient adherence to antiretroviral medication. These interventions seek to act on the behavioral target of *consistent and sustained ingestion* of medications. An entirely different behavioral target to support adherence would be an intervention to train providers to *speak and communicate* with patients more effectively and compassionately.

- - 1. **Example: behavioral target for ART initiation**

ART initiation in resource limited settings has been observed to be sluggish and incomplete, in part due to a standard of care where all patients undergo standardized pre-treatment counseling – a process that many do not complete. An implementation intervention to address this cascade gap could act on a patient behavioral target to encourage them to make a *verbal* *request of ART* from providers once they know they are eligible. Another intervention could work on a behavioral target in the providers so that they *offer* *or prescribe* ART more readily.

- 1. **Select the most prominent behavioral target if multiple are present.**

Operationally, although we will consider the behavioral target to be present if any behavioral target is explicitly stated in the manuscript, even if multiple behavioral targets could be present.

- 1. **Classify the level of the behavioral target**

Implementation interventions can act at different “levels” of influence that include the following and which we seek to document in the review. It is possible that an intervention targets multiple groups.

- - 1. Individual community members
    2. Individual patients
    3. Individual health care workers
    4. Health care organizations

Although organizations are composed of individuals, the target of behavior change may be at the level of an organization and something for which there is no individual level analog. Organizational “culture” for example may be the target of a change strategy.

- - 1. Health care systems
    2. Stakeholders and policy makers

Implementation interventions can act outside of an organizational unit but which inform the behavior of organizations and individuals. Examples of these might be legislative bodies, regulatory bodies, funding organizations or entire communities.

1. **Step 3: Identify the action target of the implementation intervention**
   1. **Implementation interventions influence behavioral targets indirectly, through changing capabilities, opportunities or motivation for the behavior that are the “action targets” for implementation interventions.**

We use Susan Michie’s “COM-B” framework to identify whether any action target is stated and whether the action target is a capability, opportunity or motivation. To illustrate the “action target,” consider the chain of events that leads to HIV testing in a particular individual in rural Uganda. First, the government launches a community based HIV testing campaign. Second, an outreach team attached to the testing campaign offers community members transportation to the campaign on a free bus. Finally, a lottery is being held at the campaign and one person who receives HIV testing will win a bicycle at the health campaign. In this example, the action target of the campaign itself is that is the intervention creates an *opportunity* for HIV testing. The action target of the free bus is the patient’s *capability* to attend the campaign. The action target of the lottery is the patient’s *motivation* to attend the campaign. There are other ways of categorizing actions, but the COM-B framework is broad and easy to apply.

1. **Step 4: Identify the action of the implementation intervention**
   1. Each action target is influenced by an “action.” Proctor suggests that an active verb be used to describe what an action is composed of. We further use the Behavioral Change Wheel as a framework for identifying and categorizing actions. These categories include actions such as “education,” “persuasion,” incentives” and others. We also abstract in a free text field a description of the action taken from the paper.
   2. Often the action can be implied even if it is not specified. For example, a study can quantify the effect of a decentralized system vs a non-decentralized system, but the implementation intervention which is how decentralization occurred, may be unspecified. The reason for specification of an action is that without how this occurred, the ability to reproduce the intervention is compromised. To be concrete, a number of studies look at integration, but some examine an effort by a ministry of health to integrate services though new co-located facilities and training new providers who can address multiple issues. Another integration intervention may occur at the level of a particular NGO-supported facility, in which a more coordinate referral system is created. These integration efforts are composed of different specific actions on the health system.
2. **Step 5: Identify the “dose” of the action.**

The dose refers to the frequency and intensity that the intervention action is applied. Just as pharmaceutical interventions must specify the dose in milligrams, the frequency of administration, and the duration of treatment, implementation interventions also report analogous parameters. For example, counseling interventions should specify, at a minimum, the duration of each sessions, the frequency that sessions are delivered, and the total number of sessions.

1. **Step 6: Identify the “temporality” of the intervention**

The relation of the intervention to underlying events should be specified. For example, with adherence interventions, specification of intervention ‘temporality’ needs to define the intervention in relation to other events such as ART initiation, lapses in care, or transfers of care to a new clinic.

1. **Step 7: Identify the actor in the implementation intervention**
   1. The actor in an implementation intervention refers to the person (or agent) that delivers the intervention.

We include characteristics of the actor as part of the specification of the actor – including their identity, selection process, background and other characteristics of human resources. We use a “low-bar” such that any specification of the actor other than the “name” is enough to quality as specification of the actor. Although we do not require specification of additional characteristics of the actor for this review, specifying the identity, selection, training and other characteristics of the actor is needed to fully understand who the actor is. For example in a peer support intervention, whether or not the peer is a person living with HIV him or herself is an important aspect of being a peer.

**Other information to be abstracted**

In addition to the Proctor dimensions, we will record publication date, title, authors, region of origin, overall effect, study design, study size, and a description of the intervention and a categorization of the intervention type.
