# Supplementary material for: Specification of implementation interventions to address the cascade of HIV care and treatment in resource-limited settings: a systematic review
Source: Implement Sci. 2017 Aug 8;12:102. doi: 10.1186/s13012-017-0630-8 (PMC5547499; doi:10.1186/s13012-017-0630-8)
Supplement: Supplementary file 4 — Emblematic studies. (DOCX 15 kb) [file 13012_2017_630_MOESM4_ESM.docx]

**Supplemental Appendix 4. Emblematic Studies**

| **First Author** | **Actor** | **Action** | **Dose** | **Temporality** | **Action or Implementation Target** | **Behavioral Target** | **Cascade Step(s) targeted by the Intervention** |
| --- | --- | --- | --- | --- | --- | --- | --- |
| de Tolly 2012 | Yes, Mobile phone company | Yes, Automatically sending text messages with either informational or motivational content | Yes, 3 or 10 text messages sent every 3 days | ??, I said ‘yes’, but perhaps they did not actually report temporality? | Yes, Capability (knowledge about testing) and Motivation | Yes, Visiting a HIV testing facility and going through testing | Testing |
| Maduka 2013 | Yes, Junior resident doctors of the department of community medicine, trained using a cited national adherence counseling training curriculum | Yes, adherence counseling plus SMS reminders | Yes, monthly counseling sessions and twice-weekly SMS messages for four months | No, the relationship between initiating this intervention and underlying events was not clear | No, no reference made to the particular behavioral determinant(s) targeted by these interventions | No, adherence was mentioned broadly, with authors stating “Adherence has been defined as the extent to which the patient continues the agreed upon mode of treatment under limited supervision”. Lack of clarity has implications for understanding the success or failure of the intervention | ART adherence |
| Pearson 2007 | Yes, patient peers who were either self- or clinician-nominated and who received one week of training on intervention delivery | Yes, home visits by patient peers to provide medication information and observe medication-taking | Yes, every weekday morning for six weeks | Yes, same day as ART initiation | Yes, Capability (provided information about medications, patient skill development), Opportunity (provided communication link between patients and providers if problems arose), Motivation (promoted psychosocial support and positive peer pressure) | Yes, daily pill-taking behavior | ART adherence |
| Hatcher 2012 | No, intervention involved a home visit by a “trained PLHA navigator”, but no information given on who this person was, how they were selected or how they were trained | No, home visits were made “in order to offer support for enrolling into HIV care” but no further detail provided about how this was performed | No, no information provided about duration or frequency of home visit attempts/visits | No, no information provided about how long after HIV testing campaign visits were made | No, no particular target described | Yes, attend one or more clinic visit for HIV care | Linkage |
